# Evaluation of the influence of electric nets on the behaviour of oviposition site seeking *Anopheles gambiae s.s*

**DOI:** 10.1186/1756-3305-7-272

**Published:** 2014-06-19

**Authors:** Sisay Dugassa, Jenny M Lindh, Steve J Torr, Steven W Lindsay, Ulrike Fillinger

**Affiliations:** 1 International Centre of Insect Physiology and Ecology (icipe), Thomas Odhiambo Campus, Mbita, Kenya; 2 University of Nairobi, School of Biological Sciences, Nairobi, Kenya; 3 Royal Institute of Technology, Stockholm, Sweden; 4 Liverpool School of Tropical Medicine, Liverpool, UK; 5 Warwick Medical School, University of Warwick, Coventry, UK; 6 School of Biological & Biomedical Sciences, Durham University, Durham, UK; 7 Disease Control Department, London School of Hygiene & Tropical Medicine, London, UK

**Keywords:** Electric net, Oviposition behaviour, Aquatic habitat, *Anopheles gambiae*, Sticky trap, Gravid trap

## Abstract

**Background:**

Electric nets (e-nets) are used to analyse the flight behaviour of insects and have been used extensively to study the host-oriented flight of tsetse flies. Recently we adapted this tool to analyse the oviposition behaviour of gravid malaria vectors, *Anopheles gambiae s.s.*, orienting towards aquatic habitats and traps by surrounding an artificial pond with e-nets and collecting electrocuted mosquitoes on sticky boards on the ground next to the nets. Here we study whether e-nets themselves affect the responses of gravid *An. gambiae s.s.*.

**Methods:**

Dual-choice experiments were carried out in 80 m^2^ screened semi-field systems where 200 gravid *An. gambiae s.s.* were released each night for 12 nights per experiment. The numbers of mosquito landing on or approaching an oviposition site were studied by adding detergent to the water in an artificial pond or surrounding the pond with a square of e-nets. We also assessed whether the supporting framework of the nets or the sticky boards used to retain electrocuted mosquitoes influenced the catch.

**Results:**

Two similar detergent treated ponds presented in choice tests caught an equal proportion of the mosquitoes released, whereas a pond surrounded by e-nets caught a higher proportion than an open pond (odds ratio (OR) 1.7, 95% confidence interval (CI) 1.1 - 2.7; p < 0.017). The separate evaluation of the impact of the square of electric nets and the yellow boards on the approach of gravid females towards a pond suggests that the tower-like construction of the square of electric nets did not restrict the approach of females but the yellow sticky boards on the ground attract gravid females to a source of water (OR 2.7 95% CI 1.7 – 4.3; p < 0.001).

**Conclusion:**

The trapping efficiency of the electric nets is increased when large yellow sticky boards are placed on the ground next to the e-nets to collect electrocuted mosquitoes, possibly because of increased visual contrast to the aquatic habitat. It is therefore important when comparing two treatments that the same trapping device is used in both. The importance of contrast around artificial habitats might be exploited to improve collections of *An. gambiae s.s*. in gravid traps.

## Background

Electric nets (e-nets) are powerful tools to evaluate the flight behaviour of insects and have been used extensively for studying tsetse flies [1-5]. Recently we adapted this method to analyse how gravid malaria vectors, *Anopheles gambiae s.s.*, orientate towards aquatic habitats [6]. This apparatus consisted of a tower of four rectangular electric nets surrounding an artificial pond, with sticky boards placed on the ground next to the e-nets to preserve all mosquitoes that get electrocuted by the nets as they approach the pond [6]. However, acknowledging that trap design affects the catch size [7-9] it is important to understand how a trap affects the behaviour of the study organism in order to interpret trapping results correctly [10,11]. This is especially important since the choice and efficacy of mosquito-trapping tools may depend on the sex, physiological state and age of the target species. Since each trap has a built-in bias, none can truly represent nature. A square of electric nets may provide a more accurate measure of attraction of an oviposition substrate than a gravid trap. This is because it would be difficult to quantify the number of mosquitoes that approach the gravid trap but never get close enough to the water surface to be trapped [12]. E-nets might, therefore, be used to quantify all mosquitoes approaching a breeding site, including those that might at the end not select the habitat to lay eggs [12,13] assuming that the trapping device does not affect the approach of mosquitoes in any way. Evidence from tsetse research using video recording shows that though extremely effective in collecting flies the tool does affect the total catch [10,11].

Another effective tool that we used recently for studying oviposition behaviour of gravid malaria vectors was an artificial pond treated with 2.5% odourless detergent [6]. Adding detergent to water reduces surface tension and hence mosquitoes landing on the water either to lay eggs or to explore the water become trapped. This simple method is very useful for quantifying the number of mosquitoes that contact a test substrate; however, it may be less suitable for understanding how mosquitoes approach water bodies. Detergent ponds are clearly much easier to set up than squares of electric nets and the question arose whether the two tools collect the same population of mosquitoes or whether there is a difference between approaching females (collected by e-nets) and landing females (collected by detergent ponds).

Previous studies suggested that the thin copper wires used for an electric net are invisible to flying insects since they readily collide with the wires [1,14-16]. However, a square of e-nets including collection boards surrounding an artificial habitat is a conspicuous structure and we therefore explored if this might impact on the orientation of gravid females. A number of studies have shown that gravid *An. gambiae s.s.* are highly sensitive to visual cues when searching for an oviposition site and landscape structures and contrasts might guide their orientation flight towards a site [17-19].

This study was intended to analyse if the presence of a functional square of e-net or any of its components (the central square of nets or collection boards on the ground) has any impact on the responses of gravid *An. gambiae s.l.* to a potential oviposition site. It also served to evaluate if the catching efficacies of a functional square of e-nets around a pond and a pond treated with detergent are similar.

## Methods

### Study site and semi-field system

The study was conducted at the International Centre of Insect Physiology and Ecology, Thomas Odhiambo Campus (icipe-TOC), Mbita (0^o^ 26′ 06.19” S, 34° 12′ 53.13”E; altitude 1,137 m above sea level), western Kenya. All experiments were implemented in a 80 m^2^ (11.4 m in length, 7.1 m wide and 4 m high) fibreglass netting-screened semi-field system under natural conditions [20]. The floor of the semi-field system was covered with sand for retention of water and creation of artificial ponds to simulate natural aquatic habitats of *An. gambiae s.s*. larvae. The relative humidity inside the semi-field system was maintained at 60–70% and the temperature ranged between 19–28°C during the experiments (17.30–08.00 h).

### Mosquito preparation

Throughout this study the Mbita strain of *An. gambiae s.s.* mosquitoes reared under controlled insectary conditions were used. This strain has been maintained at *icipe*-TOC for 13 years. Gravid mosquitoes were prepared for the experiments from the insectary colony. Three hundred female and 300 male mosquitoes, two to three days old, were kept in 30x30x30 cm netting cages and provided with 6% glucose solution *ad libitum* at 25–28°C and a relative humidity of 68–75%. Saturated cotton towels (50 × 25 cm) were folded and placed over the cages to avoid mosquito desiccation. Mosquitoes were starved from sugar for seven hours and allowed to feed on a human arm for 15 minutes at 19.00 h on the same day. Afterwards, unfed mosquitoes were removed from the cages and killed. A second bloodmeal was offered 24 h after the first. Females were kept together with males until the time of experiments 72 hours after the second blood meal. All experiments were conducted with 200 mosquitoes (visually selected as gravid based on their abdominal status) released in the centre of the semi-field system every night.

### Study design

**Figure 1:**
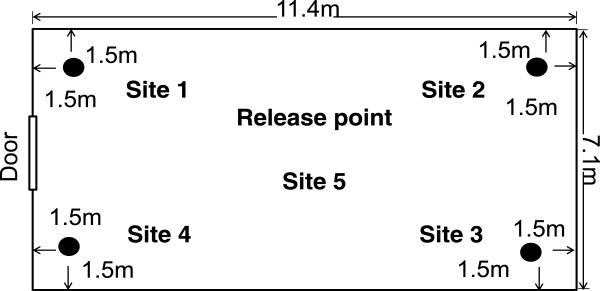
Floor plan of the semi-field system showing trap locations and mosquito release point.

Dual choice assays were used for the six experiments of this study. Definitions of all treatments used in experiments are provided in Table 1. Two artificial ponds were prepared by digging two round, black plastic bowls of 15 L capacity (36 cm diameter and 18 cm depth) into the ground in opposite corners of the semi-field system at a distance of 1.5 m from the two adjacent walls (Figure 1, Site 1–4) so that the upper lip was at the same level as the sand floor. Nine litres of tap water pumped directly from Lake Victoria were added to the bowls and 2.5% (225 ml) odourless detergent (Teepol, Chemical Industries LTD, Nairobi) was added to both ponds in all experiments. The control pond (Pond A) was an open pond (Table 1) in all six experiments whilst the treatments of the test pond (Pond B) varied between experiments. Each experiment was conducted using a randomized complete block design for the site allocation of pond A and B for 12 nights. Mosquitoes were released from the centre of the semi-field system at 17:30 h (Figure 1, Site 5) and the number of mosquitoes collected from pond A and B counted at 08.00 h the following morning.

**Table 1:** Description of the treatments used in the six experiments

**Terms**	**Description**
Pond	An artificial pond treated with 2.5% detergent (Teepol, Chemical Industries LTD, Nairobi)
Open pond	A pond that was not surrounded by any additional devices
Square of e-nets	Four e-nets joined to form a complete square around a pond including yellow collection boards placed under the nets. The nets were electrified by a 12 V battery (power source).
Tower of nets (8 mm)	Square of e-nets not connected to a power source and excluding the yellow collection boards. Inter-wire distance 8 mm.
Tower of nets (24 mm)	Square of e-nets not connected to a power source and excluding the yellow collection boards. Inter-wire distance of nets were increased from original 8 mm to 24 mm by removing every two consecutive wires.
Tower of nets (24 mm) & sticky yellow boards	Square of e-nets with inter-wire distance of 24 mm, not connected to a power source including sticky yellow collection boards.
Yellow boards	Four non-sticky yellow boards surrounding an artificial pond.

### Experiments

#### Comparison of catch rates of gravid *An. gambiae s.s* when presented with two open ponds (equal choices)

This first experiment was intended to test the hypothesis that gravid females approach the two choices in the semi-field system in equal proportions when the choices are identical. In this experiment two open ponds were set-up each night.

### Comparison of catch rates between an open pond and a square of e-nets

**Figure 2:**
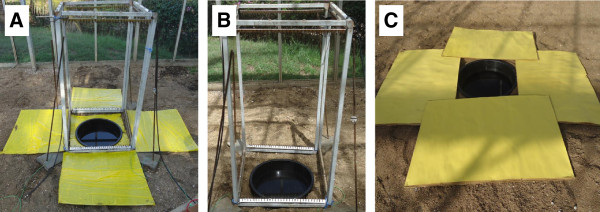
**Experimental trapping devices surrounding a pond. (A)** Square of e-nets; **(B)** Tower of nets; **(C)** Yellow boards.

The test treatment of this second experiment was a pond surrounded by a functioning square of e-nets (Figure 2A) [6]. A description of the locally made e-nets, including details of the structural design, spark box settings, current conversion and mosquito collection devices can be found in a recent methods publication [6]. Briefly, e-nets consisted of high tension copper wires stretched vertically in parallel at 8 mm distance, across an aluminium frame (1.0 m high × 0.5 m wide) with aluminium rods fixed to the two shorter opposite sides of the frame (Figure 2A). A transformer connected to a 12 v battery generated a voltage difference of >2.5 kV between adjacent wires (direct current pulsed at 50 hz) that stuns mosquitoes touching the wires. Although the voltage is high, the amperage is low (<3 amps) and hence poses no risk to humans or animals that inadvertently touch the e-net. Electrocuted mosquitoes were collected on four yellow sticky boards placed in front of each of the e-nets and in the open space inside the square of e-nets. The boards were made of 50 × 50 cm cardboard covered with polythene film coated on both sides with insect trapping adhesive (Oekotak Rollertrap, Oecos, UK). The number of mosquitoes drowned in the open pond and the sum of mosquitoes from the pond and the number stuck on the yellow boards recorded.

### Evaluation of the effect of a tower of standard nets (inter-wire distance = 8 mm) on catches of mosquitoes from an artificial pond

In the previous experiment, significantly more gravid *An. gambiae* females were collected with the square of e-nets than with the open pond. This observation could be due to a higher proportion of females approaching a pond versus landing on a pond or alternatively, could be due to the structural elements of the square of e-nets attracting gravid females to the pond. This was investigated by comparing the number of mosquitoes collected in an open pond (control pond A) with the number of mosquitoes collected in a pond surrounded by the central tower of nets (test pond B, Figure 2B). This tower of nets was not powered and it was assumed that mosquitoes flew undisturbed through the nets to reach the pond. The numbers of mosquitoes trapped in both ponds were counted.

### Evaluation of a tower of modified nets (inter-wire distance = 24 mm) on catches of mosquitoes from an artificial pond

The previous experiment showed that surrounding a pond with standard e-nets reduced the number of mosquitoes caught from the artificial pond. Consequently, we carried out an experiment to assess whether the tower of nets repelled females from the habitat or whether this observation was an artefact based on the wires creating a physical barrier to mosquito’s entry through the nets to the pond. Here, two thirds of the 8 mm spaced wires of the e-nets were removed increasing the distance to 24 mm between the adjacent wires. The intention of widening the space between the wires was to test if entry of the mosquitoes to the aquatic habitat improved. The experiment was implemented in the same way as the previous one comparing the number of females caught in the ponds.

### Evaluation of yellow boards on mosquito densities collected from ponds

Two experiments were implemented to evaluate the impact of the yellow boards on the number of gravid females caught in the pond. First, the tower of e-nets with 24 mm spacing of wires was combined with yellow sticky boards as in the original design of the squares of e-nets but the nets were not powered. This treatment around a pond was compared to an open pond by counting the number of females drowned in the two ponds. Second, an open pond (control pond A) was compared to a pond with four non-sticky yellow boards (50 × 50 cm cardboard covered with yellow cardstock paper) placed around it (test pond B; Figure 2C).

### Data analysis

Data were analysed using generalized linear mixed effects model. The analyses were done with R statistical software version 2.14.2 including the contributing packages MASS, lme4, glht, multcomp [21]. The night of experiment (same batch of mosquitoes) and location (site) where the traps were placed in the semi-field system were included in the models as random factors. The proportions of mosquitoes collected in the test treatment (pond B) per experiment (fixed factor) were modelled and the experiment with two equal choices served as reference. Models were fitted using a binomial error distribution and a logit link. The excess variation between data points (overdispersion) that remained after adjustment for all other factors was adjusted by creating a random factor with a different level for each row of the data set. The parameter estimates of the models were used to predict the mean percentages per treatment and their 95% confidence intervals (CIs) by removing the intercept from the models [22]. Multiple comparisons of treatments were also calculated based on the model parameter estimates.

### Ethical considerations

Ethical approval for this study was obtained from the Kenya Medical Research Institute’s Ethical Review Committee (Protocol no. 422).

## Results

On average 47% (95% CI 44-49%) of all released mosquitoes (n = 200) were recaptured per experimental night. The recapture rates were similar in all six experimental set ups, however, their proportional distribution between the two test treatments (Pond A and B) differed.

### Gravid *Anopheles gambiae* females respond in equal proportion to equal choices presented in a semi-field system

**Figure 3:**
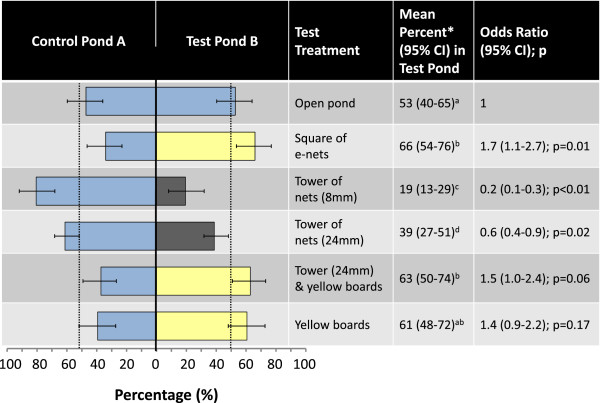
**Response of gravid female *Anopheles gambiae s.s. *towards control and test treatments.** CI = confidence interval. Multiple comparison of treatments: treatments denoted with the same letter are not significantly different.

There was no significant difference in catches from control and test ponds when they contained equal treatments. When presented with two equal choices gravid *Anopheles* females select both ponds in an equal proportion (Figure 3).

### A square of e-nets collects a higher proportion of gravid females than an open pond

The probability of a mosquito being trapped in the test was 1.7 times higher when the treatment was a functioning square of e-nets than when the treatment was an open pond (Figure 3). Two reasons could have been responsible for that and were the subject of further evaluation. Either gravid females explore aquatic habitats first before they make a decision to land or get close enough to drop eggs from flight, therefore, we would measure a larger number approaching with the e-nets than landing with the detergent pond, or the square of e-nets included structural components that make the site more attractive for gravid females.

### The tower of nets (8 mm) is not responsible for the increased approach of females towards the functional square of e-nets

It was 5 times less likely for a mosquito to be trapped in the test when the test treatment consisted of a tower of nets (8 mm) surrounding a pond than when the test treatment was an open pond (Figure 3). This result suggested either that the tower of nets repelled the gravid females from approaching the setup or was an artefact based on the physical barrier created by the wires which might have prevented most approaching females from passing through the wires to the water (Figure 3).

### The tower of nets presents a physical barrier for mosquitoes approaching a pond

Increasing the gaps between the wires of the nets significantly increased the probability of a female being collected in the pond surrounded by the tower of nets (inter-wire distance 24 mm) compared to the standard tower of nets (OR 2.6 95% CI 1.6 – 4.2; p < 0.001). However, 1.7 times fewer gravid mosquitoes were found in the test pond when the treatment was a tower of nets (24 mm) than when the treatment was an open pond (Figure 3) suggesting that the unpowered nets present a physical barrier for mosquitoes approaching the pond.

### Yellow boards increase the approach of gravid females towards an oviposition site

Inclusion of the yellow boards to the previous setup increased the probability of a female being collected in the test ponds nearly threefold (OR 2.7 95% CI 1.7 – 4.3; p < 0.001) compared to a pond without boards. This suggests that more mosquitoes were attracted towards the pond that included the yellow boards and consequently passed between the wires to reach the pond for oviposition (Figure 3).In a final experiment, non-sticky yellow boards were tested alone to assess their impact on the catch of mosquitoes from a pond. The results from this experiment were not significantly different from the experiment with equal choices, however, the confidence interval of the odds ratio suggests an association of the treatment with the outcome but we would have needed more replication to ascertain significance of the relatively small differences between the treatment arms. More importantly, however, a pond surrounded by the yellow boards alone received a similar response as a pond surrounded by a tower of nets with yellow boards and as the square of e-nets (Figure 3).

## Discussion

E-nets and detergent ponds are valuable tools for analysing the oviposition behaviour of *An. gambiae*[6]. However, our results show that they are not equivalent and hence cannot be used interchangeably. Importantly, the catch from e-nets is influenced by the conspicuous sticky boards on the ground used to retain stunned females.

The proportions of gravid females collected in two randomly allocated ponds with equal treatments over a 12 night study were similar. This indicates that two choice experiments are valid for comparing efficacies of two catching devices or oviposition substrates in our semi–field system. The square of e-nets collected about twice the proportion of mosquitoes collected in the pond treated with detergent

Surrounding a pond with the tower of nets alone was not responsible for the increased catch of females. Indeed, with the electricity switched off it was clear they presented a barrier to gravid mosquitoes. This is important, since this means it is not possible to balance the experimental design to compare approach with landing by having a square of e-nets around both ponds but connecting only one to electricity. We observed that when the e-nets were not powered the mosquitoes hit the wires while flying towards the pond and then fly off in a zigzag fashion. Torr *et al.,*[14] also suggested that the nets (0.2 mm in diameter and 8 mm apart) prevent insects from flying straight through the e-nets. The wires more likely obstruct the mosquitoes from accessing the artificial pond.

Interestingly, inclusion of the yellow sticky collection boards to the tower of e-nets (24 mm) significantly increased the number of mosquitoes that approached and passed between the wires resulting in the same proportional distribution of the catch as when the functional square of e-nets was compared with an open pond. The same result was achieved by surrounding the open pond with similar sized boards made of non-sticky yellow cardstock paper strongly suggesting that the conspicuous arrangement of the large collection boards introduced an attractive visual cue that guided more gravid females to the aquatic habitat. If the yellow colour is responsible for this or whether other colours might have resulted in the same response would need to be further evaluated but it might be more likely that the boards contributed contrast to the surrounding sand floor of the semi-field system and/or to the pond. Results from previous studies suggest that *An. gambiae s.s.* is attracted to contrasts rather than colours for oviposition [17,19,23] and that visual cues play an important role in the selection of oviposition sites [17,19,24-26]. Nocturnal mosquitoes may rely on vision especially during the early evening. This coincides with the time when the intensity of polarised light is high, a visual cue frequently used by aquatic insects [17,27,28].

Our findings appear to contradict our earlier work. Previously, we found yellow boards did not attract gravid females in contrast to shiny surfaces created by using transparent sticky foils and black sticky fibreglass nettings [6]. However, in the previous study, we only evaluated whether the gravid females respond directly to the board and actively approach the board to land on it, as they do on shiny surfaces. Clearly, they do not land on the yellow board mistaking it for a water surface as might have been the case for the other materials [17,18,26,29-31]. Here, on the other hand we compare approach to habitats surrounded by boards versus habitats without boards. In such a comparison the presence of the boards makes the habitat more attractive possibly by increasing the visual contrast around the habitat consequently helping the gravid female to localize the habitat. It may be that these boards serve as ‘oviposition site markers’ (i.e. informative of the presence of egg-laying site) [17-19] in a similar way as swarm markers have been described for mating *An. gambiae* s.s. [32].

The importance of visual cues for ovipositing mosquitoes remains poorly understood and further studies may lead to practical applications. For instance, the recently developed OviARTgravid trap [33] for sampling malaria vectors could be enhanced by adding such visual contrasts to attract more mosquitoes to the trap. This trap was designed to provide free open landing space for egg-laying mosquitoes and resembles the open ponds prepared and used in this study.

## Conclusion

The catching efficiency of a square of e-net is strongly affected by the large light-coloured collection boards. This effect of the trapping tool must be taken into consideration when designing experiments to avoid introducing collection bias. In choice experiments, both habitats must be surrounded by the same setup for mosquito collections to be comparable. When the aim is to compare the approach of gravid females to oviposition sites with landing on these sites and the e-nets on one side would not be electrified it is important to reduce the distance between the wires on those e-nets to a minimum of 24 mm to reduce the physical barrier created by those wires. Both habitat choices need to be surrounded by similar boards to make both habitats equally visual.

Whilst the attractiveness of the boards might present an obstacle in experimental studies, it could be highly beneficial for mosquito collections in the field. Conspicuous functional squares of e-nets might be an effective tool in the field to try to intercept the gravid females on their way from their resting site close to the host to the aquatic habitat. Furthermore, contrasting elements might be included in gravid traps to increase their visual attraction.

## Competing interests

The authors of this article declare that they do not have competing interests.

## Authors’ contributions

SD, JML, SJT, SWL, UF conceived the idea for this research. SD, SJT and UF developed the experimental design and protocols. SD implemented the experiments, analysed the data and drafted the manuscript. All authors contributed to the final draft, read and approved the manuscript.
